# Cement-Based Materials Modified by Colloidal Nano-Silica: Impermeability Characteristic and Microstructure

**DOI:** 10.3390/nano12183176

**Published:** 2022-09-13

**Authors:** Jie Wang, Xuesong Lu, Baoguo Ma, Hongbo Tan

**Affiliations:** 1 School of Architectural Engineering, Huanggang Normal University, Huanggang 438000, China; 2 Huanggang Ecological Architecture and Renewable Resources Research Center, Huanggang 438000, China; 3 State Key Laboratory of Silicate Materials for Architectures, Wuhan University of Technology, Wuhan 430070, China

**Keywords:** colloidal nano-silica, impermeability, mechanical properties, pore structure, interfacial transition zone

## Abstract

Colloidal nano-silica (CNS) was used to improve the mechanical and impermeability characteristics of mortar in this study. The samples were prepared with 0%, 1%, 2% and 3% (solid content) CNS addition. The mechanical strength and permeability of each mixture was studied, and the mechanism behind was revealed by hydration heat evolution, XRD, DSC-DTG, ^29^Si MAS-NMR and SEM-EDS analysis. The compressive strength and impermeability characteristics of mortars incorporating CNS were significantly improved. The experimental results demonstrated that the incorporation of CNS promoted the early hydration process of cement, thus increasing the polymerization degree of hydrated calcium silicate, decreasing the porosity, and improving the microstructure of mortar. Furthermore, 3% CNS decreased the Ca/Si ratio of the interfacial transition zone (ITZ) from 3.18 to 2.22, thus the enrichment of CH was reduced and the density and strength were improved. This was mainly because of the high pozzolanic activity of CNS, which consumed plenty of calcium hydroxide and converted to C-S-H. Besides, nanoscale CNS and C-S-H particles filled the voids between hydrates, thus refining the pore size, increasing the complexity of pores, and improving the microstructure of ITZ which contributed to the improvement of the impermeability.

## 1. Introduction

Concrete is most widely used in building engineering because of its excellent mechanical properties and low production and maintenance costs. The durability of concrete, such as shrinkage, impermeability and freeze–thaw resistance [1,2,3,4], etc., significantly affect the life cycle of buildings. The resistance to chloride ion permeability is considered as a critical indicator of concrete durability since chloride ion may accelerate the corrosion of steel bars, especially for marine concrete. Volume expansion occurs when the steel is corroded, which would lead to the cracking and deterioration of the concrete structure. Concrete cracks would in turn make chloride ions ingress into the concrete interior more easily, thus resulting in a vicious circle and decreasing the serviceability of concrete significantly.

The durability of concrete is significantly influenced by the pore structure, especially the large capillary pores, which is mainly reflected in the permeability and transport characteristics. To further improve the mechanical and durability properties, nanoparticles have been used to achieve a denser microstructure [5,6,7,8]. Unlike nano-TiO_2_ [6,9], nano-CaCO_3_ [10,11] and carbon nano-tubes [12,13], nano-silica showed high chemical reaction potential through secondary reaction with calcium hydroxide, which significantly improved the microstructure and mechanical strength [14,15,16].

Nevertheless, the easy-to-aggregate characteristic of nano-silica was still plaguing researchers, which may have decreased the mechanical strength at higher amounts [17]. Therefore, water-based colloidal nano-silica (CNS) was considered to be a suitable substitute for nano-silica powder without the risk of agglomeration [18,19]. Many scholars have carried out related research to improve the performance of cement-based cementitious materials. Kong [19] reported that the acceleration effect by incorporating CNS was more obvious in comparison with that of nano-silica powder. Chithra [20] found that when CNS was incorporated, the mechanical strength, permeability characteristics and wear resistance of copper slag concrete were greatly improved. Hou [21] also reported that CNS accelerated the setting process of fly ash–cement system. As a consequence, the strength of fly ash–cement mortar was significantly enhanced.

In view of these advances, to further clarify the mechanical and impermeability characteristics of CNS modified mortar and the microscopic mechanism behind, the hydration process, mechanical properties, pore structure, sorptivity, permeability of cement-based materials incorporating CNS were investigated in this study. Accordingly, microscopic test methods, such as hydration heat, X-ray diffraction (XRD), thermogravimetric analysis (TGA), nuclear magnetic resonance (NMR)and scanning electron microscopy (SEM) were conducted to trace the changes in hydrates and microstructure of different samples. Fractal dimension of pore surface was also used to quantitatively analyze the inhomogeneity and complexity of pore structure in different samples.

## 2. Experimental

### 2.1. Raw Materials

**Figure 1:**
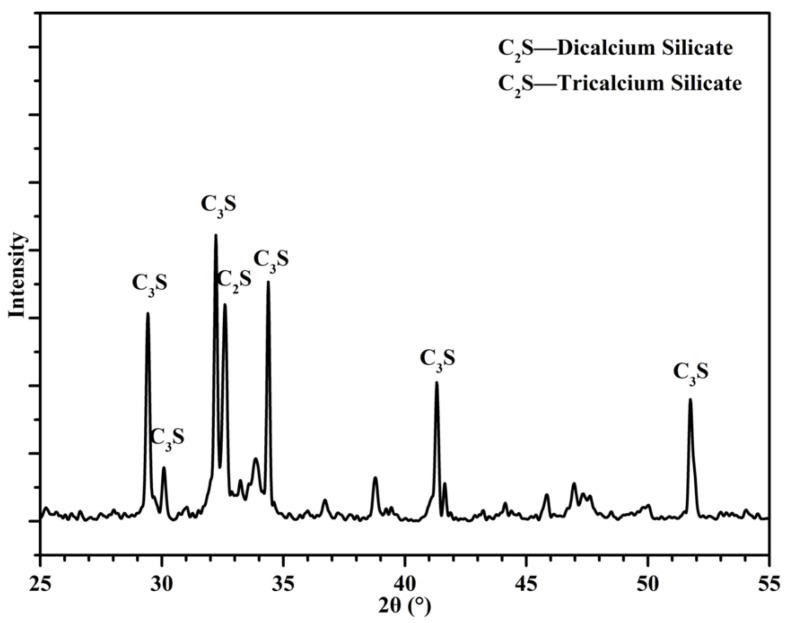
Mineral composition of cement.

**Figure 2:**
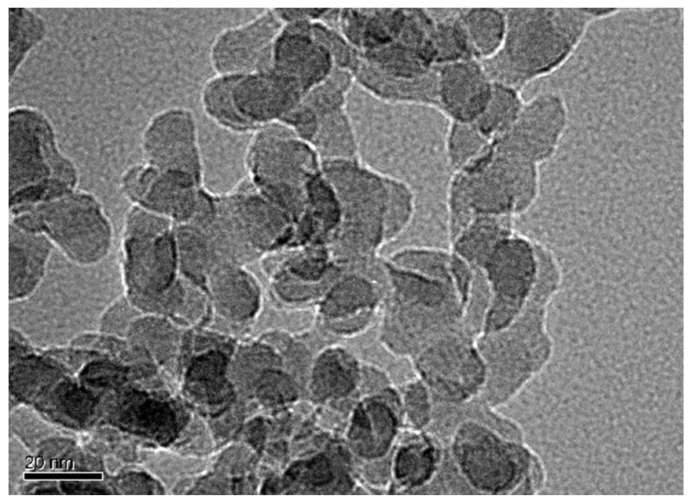
TEM image of CNS.

Portland cement (CEM I 52.5) and CNS with 40% solid content (average particle size: 14 nm) were used in this study. The phase constitution of cement was characterized by XRD, and the microstructure of CNS was characterized by TEM, as seen in Figure 1 and Figure 2. The chemical component of cement is given in Table 1. The physical characteristics of CNS are presented in Table 2. The samples were prepared with standard sand and tap water in accordance with Chinese standards.

### 2.2. Sample Preparation for Strength Test and Microscopic Analysis

The mix proportions of mortar and paste are shown in Table 3. It is noticed that all the CNS added was recorded as the solid content. The water-to-binder ratio was kept at 1/2 and the binder-to-sand ratio was kept at 1/3 in this study. CNS was added at the content of 0%, 1%, 2% and 3% by mass of binder. Superplasticizer was used in the mixture to ensure sufficient workability. Mortar samples were prepared in accordance with GB/T 17671-1999 and molded with a size of 40 × 40 × 160 mm^3^ for compressive testing. All paste samples for XRD, DSC-DTG, ^29^Si NMR and SEM testing were molded with a dimension of 40 × 40 × 40 mm^3^. Terminate the hydration process of samples with anhydrous ethanol when cured to a specified time. Then dried the samples at 40 °C for 24 h in a vacuum drying oven. Fragmented sample was used for microscopic morphology analysis; and the remaining sample was ground to pass 75 μm sieve for hydration product analysis.

### 2.3. Test Methods

The mechanical strength of the sample was tested based on Chinese national standard GB/T 17671-1999. Three 40 × 40 × 160 mm^3^ samples were employed for the mechanical strength test. The mechanical strength of the mortar was tested by a WYA-300 automatic testing machine, in which the flexural strength was tested at the loading rate of (50 ± 10) N/S and the compressive strength was tested at the loading rate of (2.4 ± 0.2) KN/S.

Mortar samples were prepared with a dimension of Φ100 mm × 50 mm and cured in a humidity chamber for 28 days. Then the samples were immersed in water for 2 days and the wet weight was referred to as M0. Finally, the samples were placed in a vacuum oven at 50 °C for 3 days to achieve constant weight (*M_1_*). The total water absorption of mortar was determined by Equation (1):

(1) $$Water\;absorption=\frac{M_{0}-M_{1}}{M_{1}}\times 100\%$$

**Figure 3:**
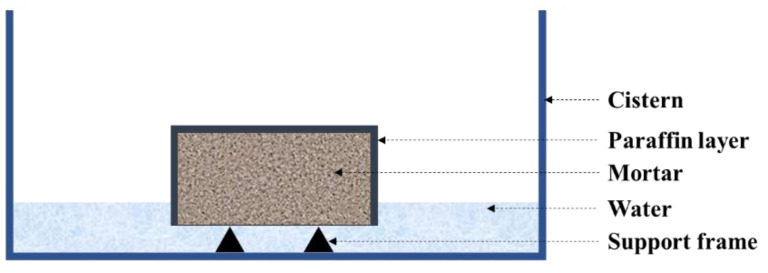
Schematic diagram of capillary water absorption test.

Then, we sealed the surface of the sample with paraffin except the surface in contact with water (Figure 3). The weight of each sample was tested at different time nodes up to 7 days. The sorptivity coefficient (*S*) can be calculated according to the Equation (2):

(2) $$I=\frac{\Delta M}{A\times \rho }=S\times \sqrt{t}$$

where *A*—the area of specimen immersed in water, mm^2^; *ΔM*—the weight of absorbed water, g; *S*—the sorptivity coefficient of mortar, mm/min^1/2^; *ρ*—the water density, g/cm^3^; *t*—the absorption time, min.

**Figure 4:**
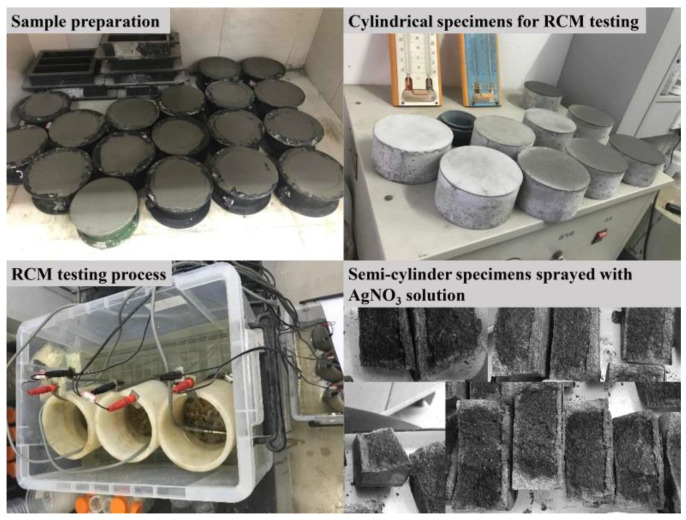
Experimental process of RCM test.

**Figure 5:**
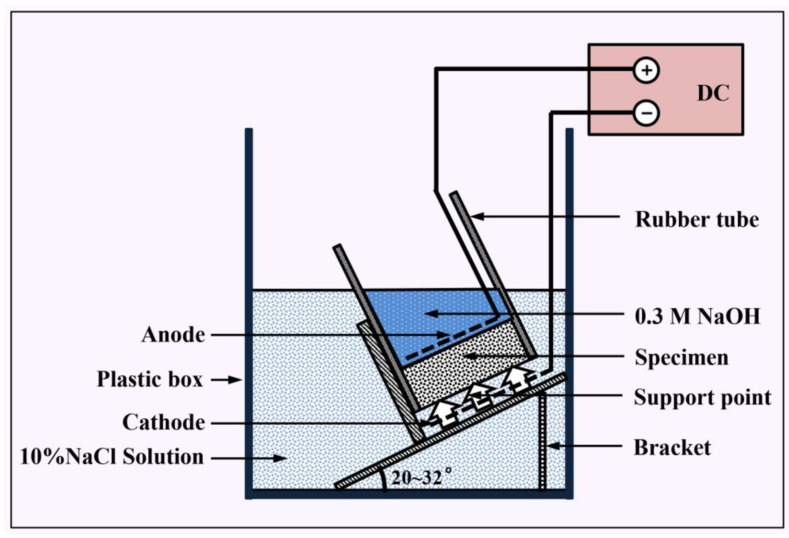
Schematic of RCM test setup.

The experimental setup for rapid chloride migration (RCM) test was shown in Figure 4 [22], which was carried out according to Chinese standard GB/T 50082-2009. The mixes for the RCM test were firstly cast into Φ100 mm × 50 mm molds and the test was performed on the sample cured for 28 days. Firstly, we placed the samples in a vacuum container for vacuum processing for 3 h. Afterwards, the samples were immersed into saturated lime water for 18 ± 2 h. After completing the above steps, we installed the test device according to Figure 5. Then a 30 ± 0.2 V external electrical potential was applied to the upper and lower sections. After the migration test, samples were rinsed clean and then split into two semi-cylinders along the axial direction. Finally, 0.1 mol/L AgNO_3_ solution was sprayed on the cross section, and the penetration depth of the samples was measured at 10 different locations. Three samples were used for each test group. The *D_RCM_* was calculated as Equation (3):

(3) $$D_{RCM}=\frac{0.0239\times \left(273+T\right)L}{\left(U-2\right)t}\left(X_{d}-0.0238\sqrt{\frac{\left(273+T\right)LX_{d}}{U-2}}\right)$$

where *D_RCM_*—chloride diffusion coefficient, m^2^/s; *U*—the applied voltage, V; *T*—average temperature, °C; *L*—the height of sample, mm; *X_d_*—penetration depth, mm; *t*—test time, h.

An isothermal conduction calorimeter was used for hydration heat test. The test time lasted 72 h and the temperature kept at 20 °C.

X-ray diffraction analysis was employed to characterize the phase composition of the samples. The scanning range was from 5° to 60°and the scanning speed kept at 10°/min.

A spectrometer (Bruker Avance III, Billerica, MA, USA) was used in ^29^Si MAS-NMR analysis. Cement reaction degree (*α_PC_*) and average chain length (*ACL*) of C-S-H which represented the polymerization degree were calculated according to Equations (4) and (5) [23,24]:

(4) $$\alpha_{PC}=1-\frac{I\left(Q^{0}\right)}{I\left(Q^{0}\right)+I\left(Q^{1}\right)+I\left(Q^{2}\right)}\;\;$$

(5) $$ACL=\frac{2I\left(Q^{1}\right)+2I\left(Q^{2}\right)}{I\left(Q^{1}\right)}\;$$

where *I*(*Q*^0^), *I*(*Q*^1^) and *I*(*Q*^2^) represented the integrated intensities of signals *Q*^0^, *Q*^1^ and *Q*^2^ in hydrated cement sample, respectively.

DSC-DTG analysis was performed on NETZSCH STA 449C, Selb, Germany. The heating rate of this test was kept at 10 °C/min and the test temperature range was 35 °C to 1000 °C.

A mercury intrusion porosimetry was used to characterize the pore structure of the sample. The maximum testing surface tension was 480 MPa.

A scanning electron microscope was used to characterize the microstructure of hydrated cement sample.

## 3. Results and Discussion

### 3.1. Permeability Characteristics

#### 3.1.1. Total Water Absorption

**Figure 6:**
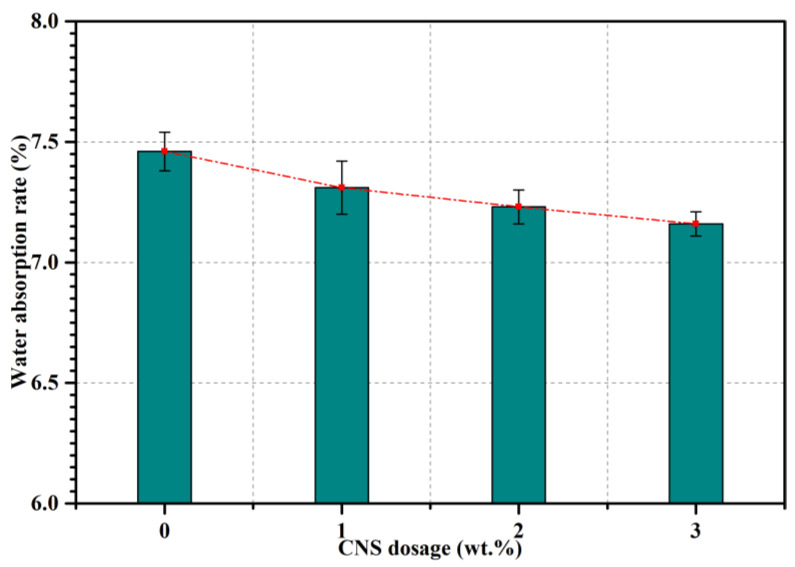
Water absorption of mortars.

Water absorption was used to characterize the water permeability of samples in this study, which was one of the criteria reflecting the porosity of mortar and concrete. The test results of mortar incorporating CNS are presented in Figure 6. The total water absorption rate, to a certain degree, can reflect the open porosity and compactness of mortar. High water absorption rate may lower the durability of cement mortar and concrete since most of the harmful solute and ions were penetrated into the interior of mortar and concrete through the pores along with water [25]. The water absorption rate was observed to decrease when CNS was incorporated in this study and the lowest water absorption rate value was noted in the specimen containing 3% CNS (N3), which was decreased by 4.02% in comparison with sample C0. It was inferred that CNS decreased the water absorption of mortar and the higher the dosage of CNS, the lower the water absorption was observed.

#### 3.1.2. Capillary Water Absorption

**Figure 7:**
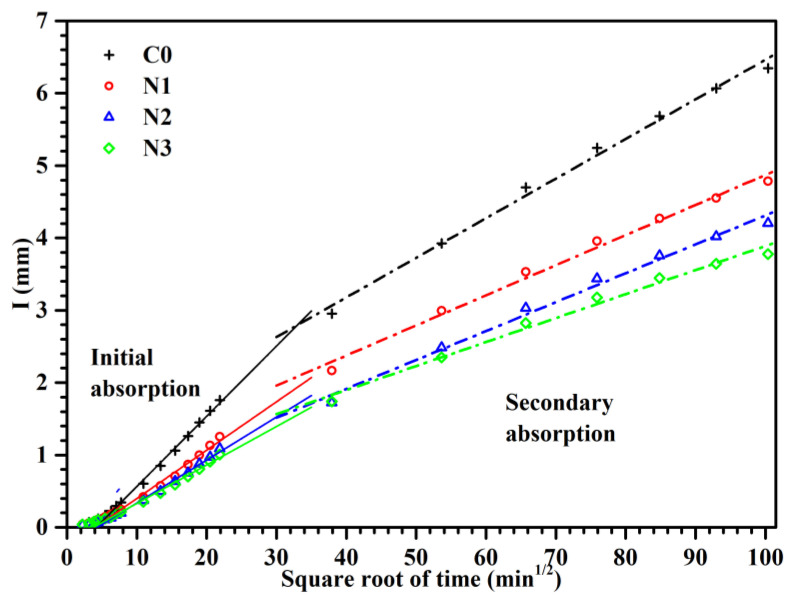
Capillary water absorption test results.

The sorptivity is also an important indicator to reflect the transport characteristics, which can characterize the absorption and transport behavior of water by porous materials through capillary channels. As seen in Figure 7, the relationship between the cumulative water absorption (I, mm) of mortar and the square root of elapsed time (*t*^1/2^, min^1/2^) was plotted. Among them, the slope of the curve from 1 min to 6 h was recorded as the initial sorptivity coefficient (S_i_), and the slope between 1 d to 7 d was recorded as the secondary sorptivity coefficient (S_S_) [26]. As previous literature reported [27], since water would encounter much smaller pores (such as gel pores) at later absorption periods when penetrated into the interior of mortar, thus the secondary sorptivity coefficient was much lower than that of the early absorption period. For another reason, plenty of gel pores existed in the interface transition zone of the aggregate. The stable or sub-stable form of water and air would be formed at the interface when water invaded the pores, which would definitely hinder the adsorption rate.

The initial and secondary sorptivity coefficient of plain cement and CNS modified mortar at 28 days are shown in Table 4. It was obvious that both S_i_ and S_S_ were decreased with the incorporation of CNS to some extent. Taking sample N3 as an example, the initial sorptivity coefficient decreased by 45.36% and the secondary sorptivity coefficient decreased by 38.89% in comparison with control sample (C0), respectively. It is well known that water migrates mainly through the capillary pores in cement-based materials. Therefore, it was inferred that the incorporation of CNS promotes the densification of microstructure and reduces the porosity of mortar, especially the connectivity of pores.

#### 3.1.3. Chloride Diffusion Coefficient

**Figure 8:**
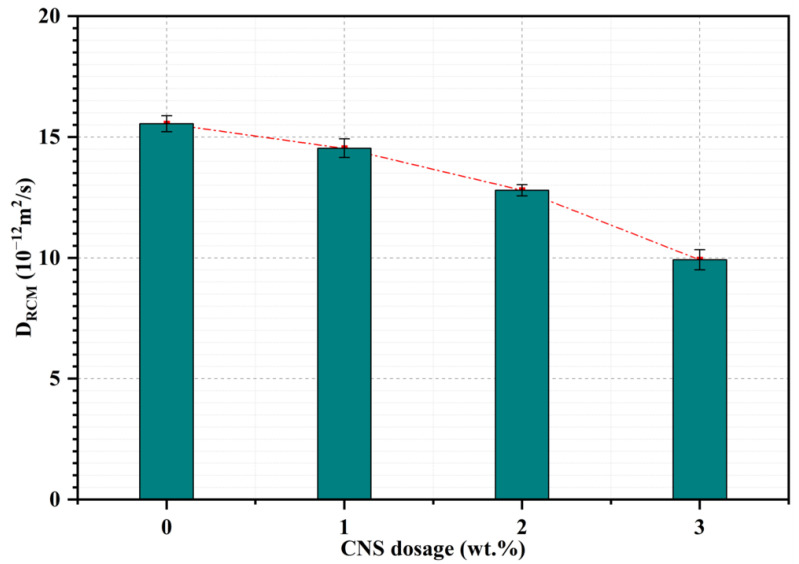
Chloride diffusion coefficient of mortars at 28 days.

The D_RCM_ of mortars at 28 days incorporating different dosage of CNS was investigated. It can be seen from Figure 8 that samples without CNS addition gained the highest D_RCM_ value, but when CNS was incorporated the D_RCM_ showed a downward trend. The D_RCM_ of mortar with 1%, 2% and 3% CNS were 14.54 × 10^−12^ m^2^/s, 12.79 × 10^−12^ m^2^/s and 9.92 × 10^−12^ m^2^/s, which were decreased by 6.5%, 17.7% and 36.2% in comparison with the control sample (15.55 × 10^−12^ m^2^/s), respectively. This result implied an excellent enhancement of CNS on the chloride penetration resistance. As mentioned above, because nano-silica had an ultra-high chemical reaction activity, plenty of additional C-S-H was generated in the samples with CNS addition. Therefore, the interconnected pores were blocked, the total pore volume was reduced and the complexity of pores was increased [28]. Consequently, the impermeability of cement mortar was significantly improved.

**Figure 9:**
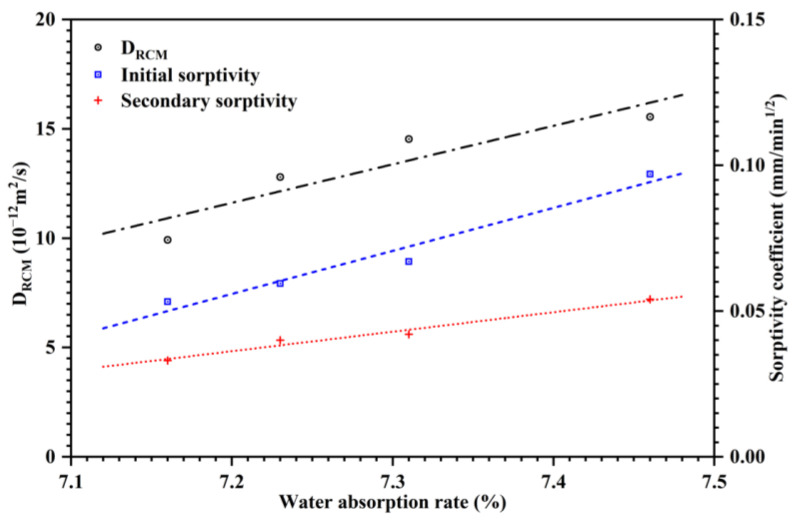
The relationship between sorptivity coefficient, water absorption rate and chloride diffusion coefficient of mortars.

To clarify the relationship between water absorption, sorptivity coefficient and D_RCM_, the correlation between them was established as shown in Figure 9. The high value of the determination coefficient (R^2^(D_RCM_) = 0.77, R^2^(S_i_) = 0.94, R^2^(S_s_) = 0.96) indicated the strong correlations between these indicators. It was observed from the figure that the trend both in D_RCM_ and sorptivity of mortars was to increase with water absorption rate, which was related to the dosage of CNS.

### 3.2. Mechanical Strength

**Figure 10:**
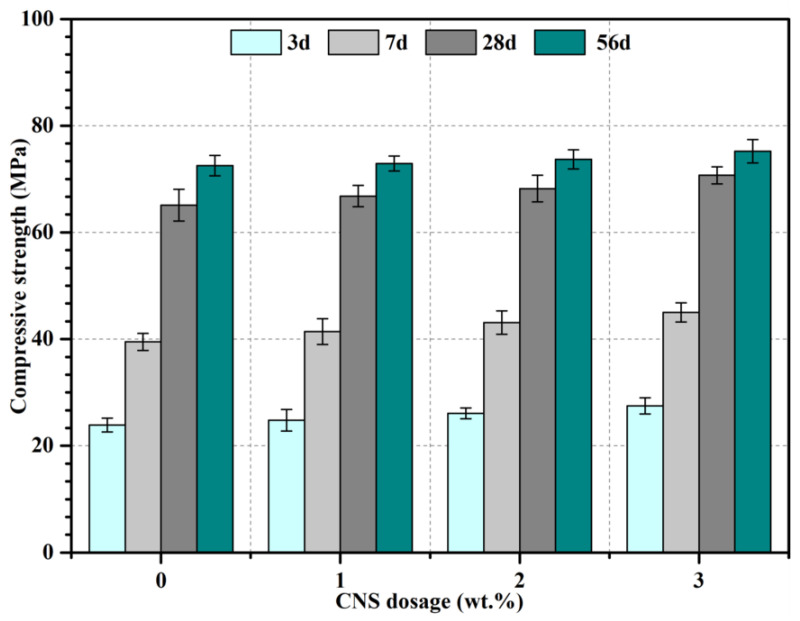
Compressive strength of cement mortars containing CNS.

As can be seen from Figure 10, due to the continuous hydration of cement clinker, the compressive strength of mortar increased with curing time. In addition, it was obvious that the strength increased significantly with CNS dosage up to 3%. For instance, the 28-d strength increased by 2.6%, 4.8% and 8.6% when 1%, 2% and 3% CNS was incorporated, respectively. It was inferred that additional low alkaline C-S-H gels with higher strength and better stability were generated because of the high reaction activity of CNS [29], with contribution to the strength. Besides, nano-silica and additional C-S-H particles acted as a crystallization nucleation site, which accelerated the hydration of cement and improved the microstructure of the matrix thus increasing the mechanical strength considerably [30,31].

### 3.3. Pore Structure Analysis

**Figure 11:**
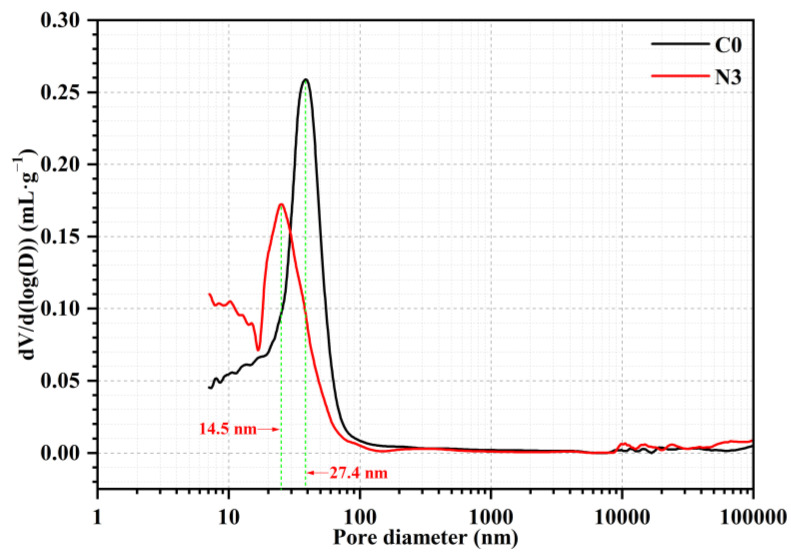
MIP results of 28 d cured samples C0 and N3.

Based on the aforementioned research, samples C0 and N3 were chosen for pore structure analysis. As shown in Table 5, 3% CNS decreased the sample porosity from 0.1163 mL·g^−1^ to 0.1103 mL·g^−1^. Furthermore, the volume of pore < 25 nm increased when CNS was incorporated, whereas the volume of pore ≥ 25 nm was decreased to a certain degree. Among them, many pores between 10 and 100 μm existed in samples C0 and N3, with the possible reason being the insufficient vibration during molding process. The most probable pore-size of sample N3 was reduced from 27.4 nm to 14.5 nm (Figure 11), implying the refinement of pore structure by incorporating CNS. This result further confirmed the improvement effect of microstructure by incorporating CNS, which was consistent with the strength and permeability analysis.

The fractal dimension of pore surface could effectively reflect the inhomogeneity and complexity of pore structure. According to Zhang [32], the following relationship existed between the cumulated intrusion work *W_n_* and the cumulated mercury intrusion surface *Q_n_* (Equation (6)). Among them, *W_n_* and *Q_n_* could be calculated according to Equations (7) and (8).

(6) $$\mathrm{lg}\left(W_{n}\right)=\mathrm{lg}\left(Q_{n}\right)+C$$

(7) $$W_{n}=\overset{n}{\underset{i=1}{\sum }}P_{i}\Delta V_{i}$$

(8) $$Q_{n}=r_{n}^{2-D}V_{n}^{D/3}$$

where *P_i_* was the test pressure; *V_i_* was the intruded volume of mercury; *r_n_* was the pore radius; *C* was a constant.

Finally, Equation (6) could be rewritten as the following Equation (9). *D* represented the surface fractal dimension of pore surface.

(9) $$\mathrm{lg}\left(\frac{W_{n}}{r_{n}^{2}}\right)=D\mathrm{lg}\left(\frac{V_{n}^{1/3}}{r_{n}}\right)+C$$

**Figure 12:**
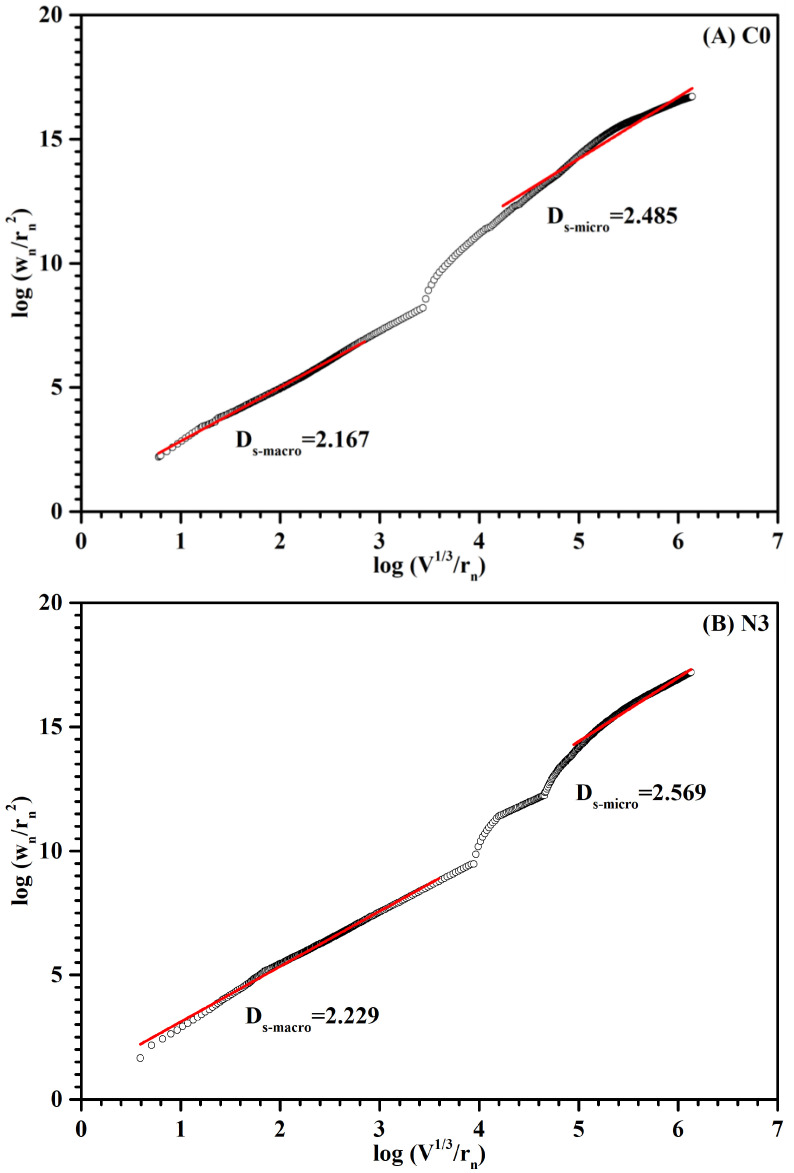
The relation between W_n_/r_n_^2^ versus V_n_^1/3^/r_n_ for sample (**A**) C0 and (**B**) N3 at 28 days.

Fractal dimension reflected the effectiveness of complex bodies occupying space, and it was an important measurement for the irregularity of complex bodies. According to the fractal theory, the numerical distribution of fractal dimension was between 2 and 3. The bigger the fractal dimension value, the higher the complexity of the pore structure [33,34]. In this study, three fractal regions were observed in Figure 12: macro-region, transition-region and micro-region [35]. Macro-region was the main pore size range which affected the impermeability, corresponding to the large capillary pores; micro-region was mainly related to the pore structure of C-S-H while the transition region was more relevant to the smaller capillary pore between the space of hydration products. As shown in Figure 12, the D_s-macro_ and D_s-micro_ of sample C0 were 2.167 and 2.485, which were lower than that of sample N3 (D_s-macro_ = 2.229, D_s-micro_ = 2.569). The result implied that the incorporation of CNS enhanced the inhomogeneity and complexity of the pore structure of paste samples.

In summary, CNS improved the pore structure considerably: many of the micro pores converted into micro pores and the complexity of the spatial distribution of pores was increased as well. For this reason, the microstructure of the matrix was significantly optimized, thus decreasing the possibility of water and external harmful ions (such as Cl^−^, SO_4_^2−^, etc.) penetrating the interior of cement-based materials, which was one of the reasons for the decease of sorptivity and chloride diffusion coefficient of mortar.

### 3.4. Analysis of Hydration Process

#### 3.4.1. Hydration Heat Evolution

**Figure 13:**
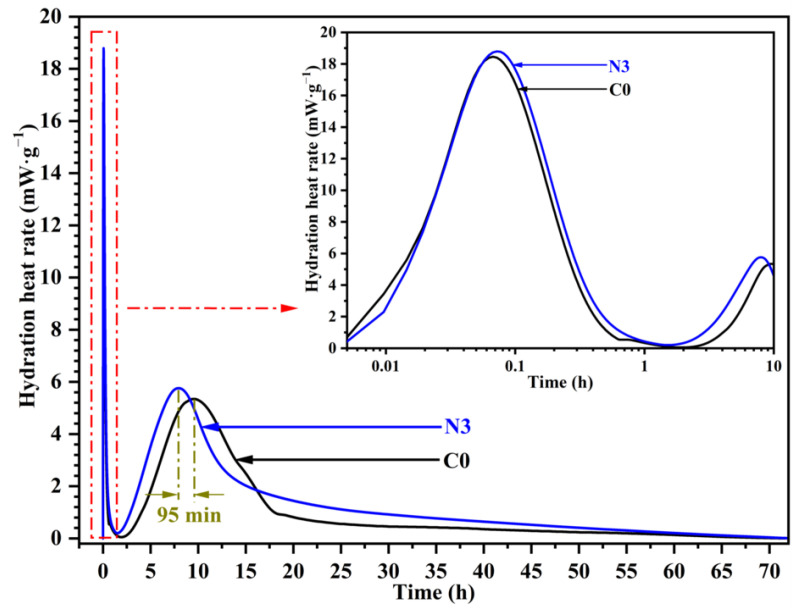
Rate of heat evolution of different samples.

Hydration heat was used to characterize the early hydration process. Figure 13 presents the hydration heat evolution process of samples. Two main exothermic peaks at different hydration stages were observed on the hydration exothermic curve. The exothermic peak caused by the formation of AFt appeared within 1 h. There was not any significant difference between the pastes with and without CNS addition during this period. The second exothermic peak due to the hydration of C_3_S appeared at ca. 8 to 10 h. It was observed that the induction period was significantly shortened when CNS was added. The position of the second exothermic peak of the sample with 3% CNS addition appeared 95 min in advance compared to C0. The intensity of second exothermic peak also showed 9.6% higher than C0 sample. Moreover, the rate of heat evolution of paste with 3% CNS was always higher than that of C0 during induction period.

**Figure 14:**
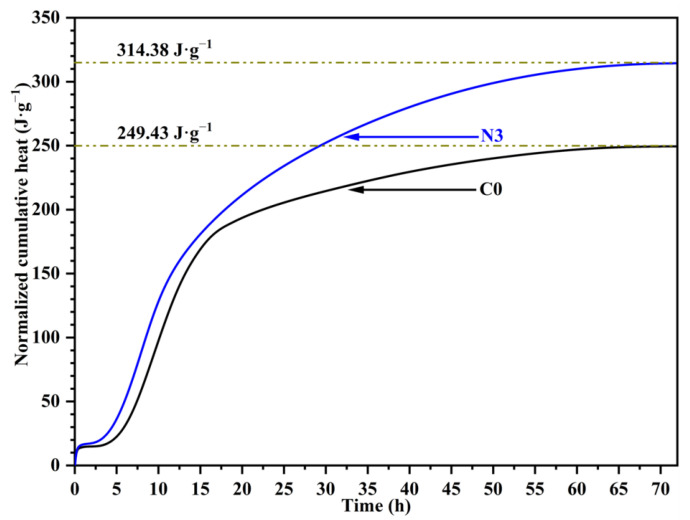
The accumulated heat of different samples.

The accumulated exothermic heat of different cement pastes is illustrated in Figure 14. The total exothermic heat of the sample with 3% CNS increased by 26.0% compared with sample C0 at 72 h. The results indicated that the hydration process was accelerated at an early stage by incorporating CNS. This phenomenon could be attributed to the acceleration effect of nano-silica, which provided nano-scale nucleation sites for hydrates [36]. In addition, lots of Ca^2+^ ions were adsorbed by nano-silica particles, thus decreasing the concentration of Ca^2+^ ions. Accordingly, based on the equilibrium theory of ion concentration, the dissolution rate of calcium silicate and the cement hydration rate would be increased more effectively [20], which further promoted the hydration process.

#### 3.4.2. XRD and Thermal Analysis

**Figure 15:**
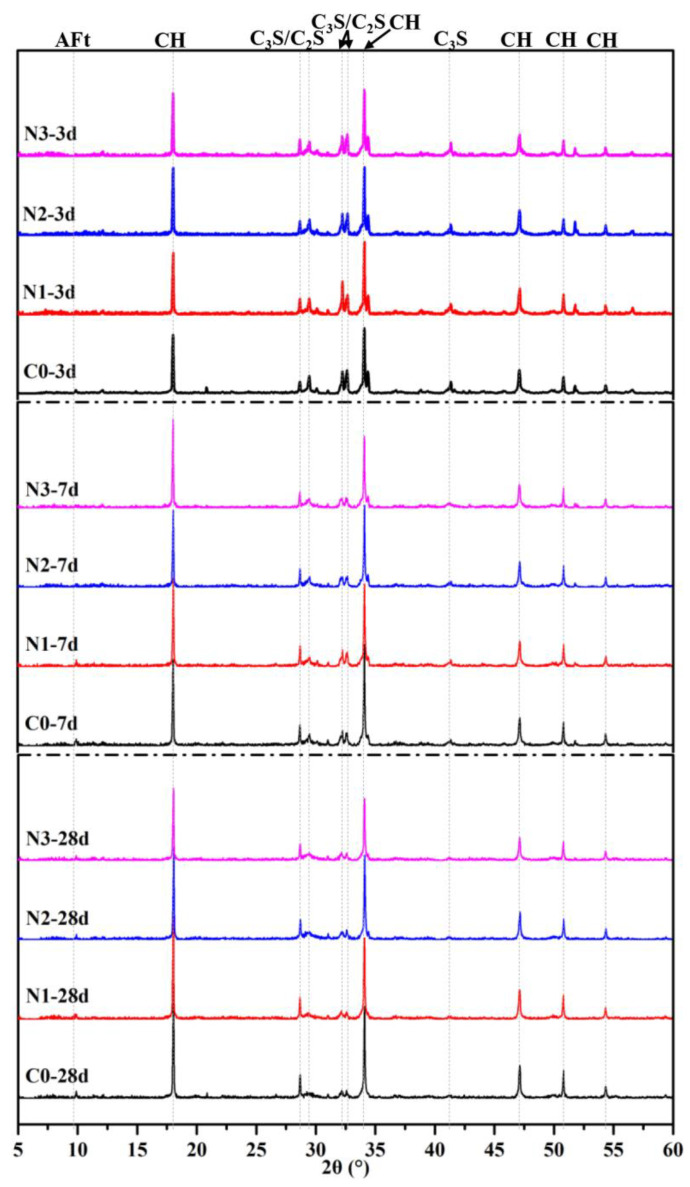
XRD curves of different cement pastes.

Figure 15 shows the XRD patterns of each sample at different curing age. Portlandite (CH), dicalcium silicate (C_2_S) and tricalcium silicate (C_3_S) diffraction peaks were observed in the patterns. The diffraction peaks of C_3_S and C_2_S decreased with curing time, implying the ongoing hydration of cement clinker. The intensity of CH diffraction peaks did not show any significant difference when CNS was incorporated at 3 days. However, when the hydration reaction proceeds to 7 days, the intensity of CH diffraction peaks of cement paste with CNS addition showed a drop compared to C0. As a typical hydration product, the content of calcium hydroxide in the samples could be used to characterize the hydration process and evaluate the secondary reaction activity of CNS to a certain extent. Therefore, CH content can be considered as the main indicator of the hydration process in blended cement paste.

**Figure 16:**
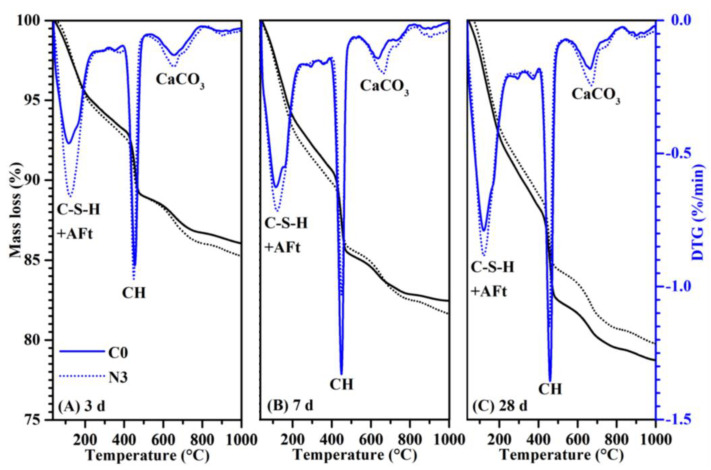
TG-DTG curves of different cement pastes at (**A**) 3 days, (**B**) 7 days and (**C**) 28 days.

In order to quantitatively analyze the CH content of samples with or without CNS, DSC-DTG analysis was conducted and the CH content of samples was calculated according to Equation (10) (Figure 16) [37].

(10) $$CH\left(\%\right)=ML_{CH}\times \frac{M_{CH}}{M_{H}}+ML_{CC}\times \frac{M_{CH}}{M_{C}}=ML_{CH}\times \frac{74}{18}+ML_{CC}\times \frac{74}{44}$$

where, *M_CH_*, *M_H_* and *M_C_* represent the molar weight of Ca(OH)_2_, H_2_O and CO_2_; *ML_CH_* and *ML_CC_* represent the percentage of weight loss corresponding to the pyrolysis of Ca(OH)_2_ and CaCO_3_.

**Figure 17:**
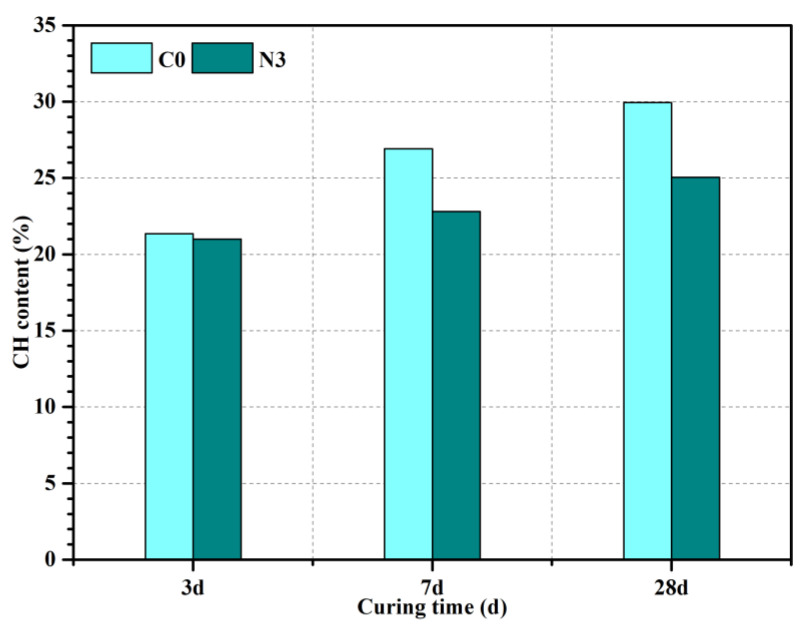
CH content of different cement pastes.

TG curves showed the mass loss of different cement pastes, and DTG curves were used to find the temperature range for the decomposition of CH and CaCO_3_ (carbonized by CH). In Figure 16, the endothermic peak at 50–200 °C was caused by the dehydration of C-S-H and AFt; the endothermic peak at 400–550 °C was caused by the CH dehydration; and the endothermic peak at 650–800 °C corresponded to the CaCO_3_ pyrolysis. As seen in Figure 17, in the samples cured for 3 days there was no significant difference in CH content between the 3% CNS added sample and the C0 sample. As the hydration reaction continued, the CH content of N3 at 7 days and 28 days decreased by 15.2% and 16.4% compared to that of C0, respectively. This was because nano-silica could promote the hydration process at an early stage and more CH was generated during this process. Meanwhile, a large amount of CH was consumed because of the ultra-high pozzolanic reactivity of nano-silica. After 3 days, the acceleration effect was gradually weakened, and most of the nano-silica was transformed to C-S-H by secondary reaction. This result further demonstrated the enhancement of CNS on early hydration of cement-based materials, which helped to form a denser microstructure and thus improved the mechanical properties.

#### 3.4.3. ^29^Si MAS-NMR Analysis

**Figure 18:**
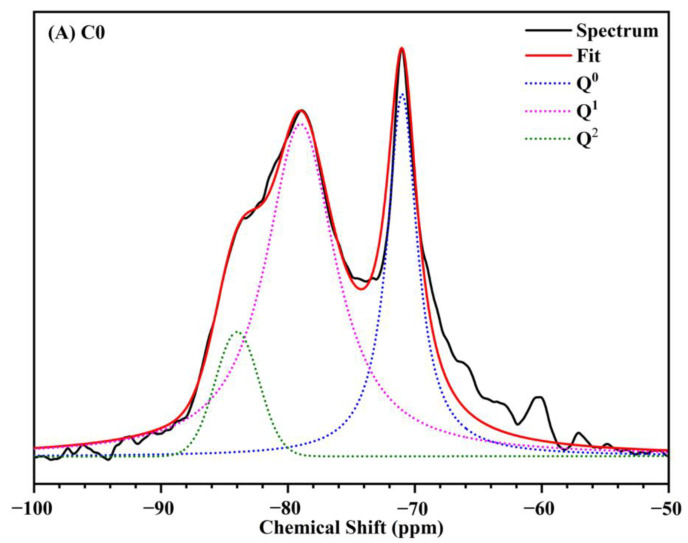

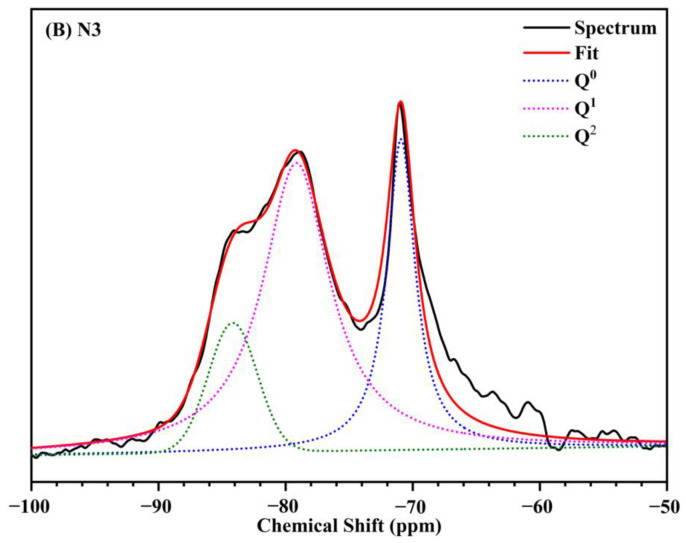
The deconvolution of ^29^Si NMR spectrum of (**A**) C0 and (**B**) N3 at 28 days.

To further study the hydration process and hydrates of different samples, ^29^Si MAS-NMR analysis was used in this research. The hydration products of C0 and N3 were tested by ^29^Si NMR (Figure 18A,B), respectively. Then, α_pc_ and ACL were calculated according to the Equations (4) and (5), respectively (Table 6).

Several researchers [23,38] have demonstrated that Q^0^, Q^1^ and Q^2^ are corresponding to different types of SiO_4_ tetrahedra, in which Q^0^ represents the silicate mineral in cement, Q^1^ and Q^2^ represent the transition from calcium silicate to C-S-H. Peak positions corresponding to Q^0^, Q^1^ and Q^2^ were located at around −70 ppm, −79 ppm and −84 ppm, respectively [39]. Q^0^, Q^1^, and Q^2^ peaks were clearly detected in both C0 and N3 samples cured for 28 days (Figure 18). As shown in the quantitative analysis results in Table 6, the relative intensity of Q^0^ and Q^1^ were decreased and the relative intensity of Q^2^ was increased when 3% CNS was incorporated. The hydration degree of the 3% CNS-added sample at 28 d was 72.72%, which was 3.22% higher than that of pure cement paste (70.45%). Meanwhile, 3% CNS increased the ACL value of the sample by 4.74%, contributing to a higher silicate polymerization degree, which would lead to a more compact microstructure with higher mechanical properties [40], in agreement with the aforementioned analysis.

### 3.5. Microstructure Analysis

#### 3.5.1. Microscopic Morphology

**Figure 19:**
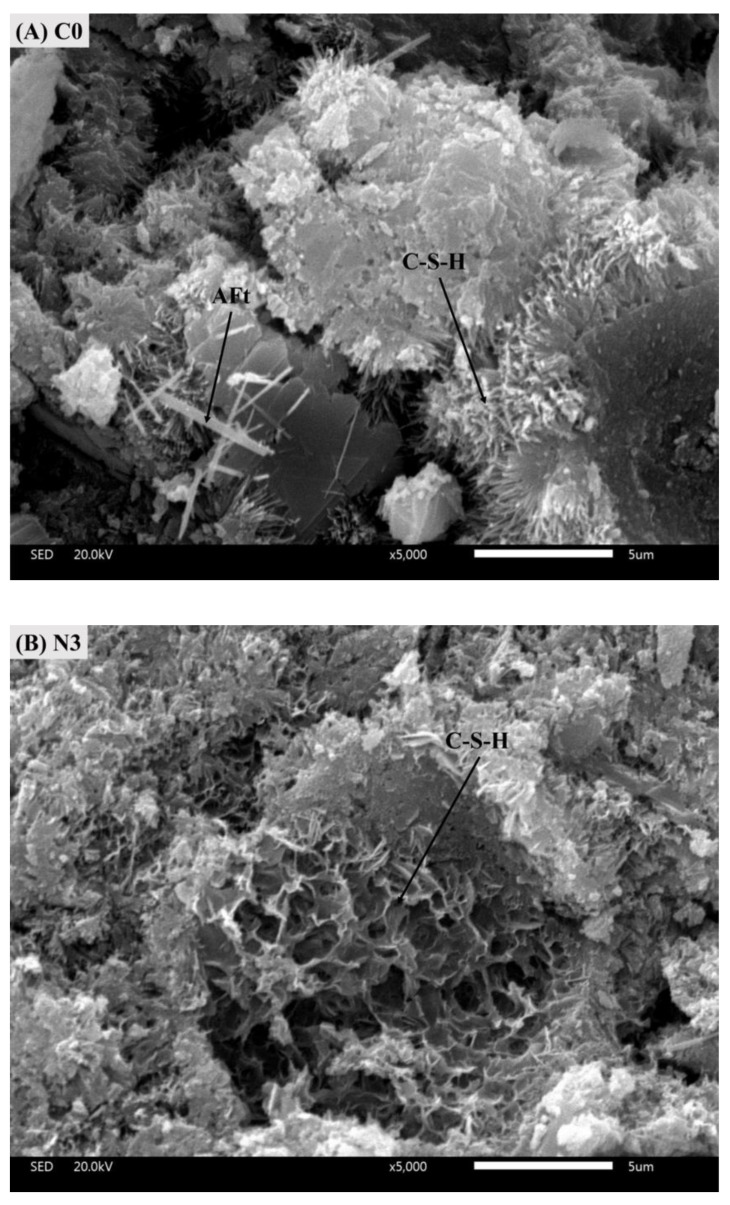
SEM images of (**A**) C0 and (**B**) N3 at 28 days.

To verify the mechanism of CNS on the cement hydration analyzed above, SEM measurements were performed in this study (Figure 19). A relative loose structure with many voids was observed in sample C0 after 28 days and large amounts of fibrous C-S-H was clearly observed. A small amount of needle-like ettringite (AFt) crystals were embedded between the hydrates. Nevertheless, the C-S-H gels in sample N3 exhibited a sponge-like structure and were closely connected to other hydrates in which a much denser microstructure with less pore was observed.

#### 3.5.2. ITZ Analysis

The interfacial transition zone (ITZ) was generally considered to be the weakest area in concrete because of the porous microstructure and the enrichment of CH [41], which has a great negative impact on strength and durability. The composition of ITZ was complex since the variety of kinds of hydrates including C-S-H gel, CH crystal and small amount of AFt crystal were interspersed with each other. Therefore, Ca/Si ratio analysis could be employed to indirectly characterize the enrichment and crystallization growth of CH in ITZ. As previous research reported, the normal Ca/Si ratio range of C-S-H gel is 1.2–2.3 [42]. The higher the Ca/Si ratio value, the higher the degree of CH enrichment, which is obviously not conducive to the development of concrete properties. In this case, BSE-EDS analysis was used to conform the improvement of CNS on the ITZ which was beneficial to the mechanical strength and impermeability characteristics of mortar. 

**Figure 20:**
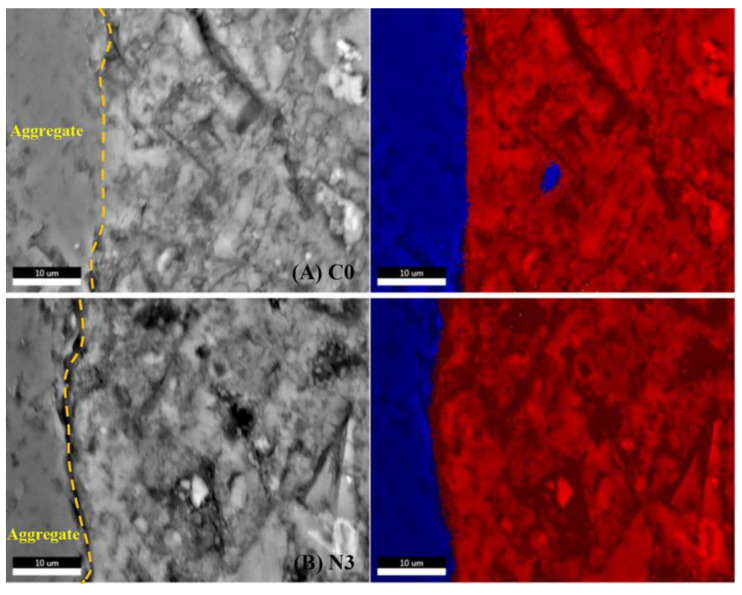
BSE-EDS analysis of ITZ of (**A**) C0 and (**B**) N3 at 28 days.

As shown in Figure 20A,B, two main areas (blue and red) can be observed in the images. The blue area represents the aggregate in mortars which should be excluded when calculating Ca/Si ratio; the red area represents the ITZ composed of variety kinds of hydration products. As shown from the results of element analysis of ITZ listed in Table 7, the incorporation of 3% CNS decreased the Ca/Si ratio of ITZ in the sample C0 by 30.2%, which was reduced from 3.18 to 2.22. It can be inferred that CNS significantly improves the microstructure of ITZ and this was considered as one of the reasons for improving the mechanical strength and impermeability. The experimental results can be explained by two main reasons: firstly, the secondary reaction of CNS with ultra-high pozzolanic activity consumed a lot of CH and restricted its crystallization growth; secondly, large amounts of CNS and C-S-H particles occupied the available voids in ITZ, thus further reducing the size of CH crystals. This result also reveals the improved mechanical and impermeability properties of CNS modified mortars.

### 3.6. Mechanism

**Figure 21:**
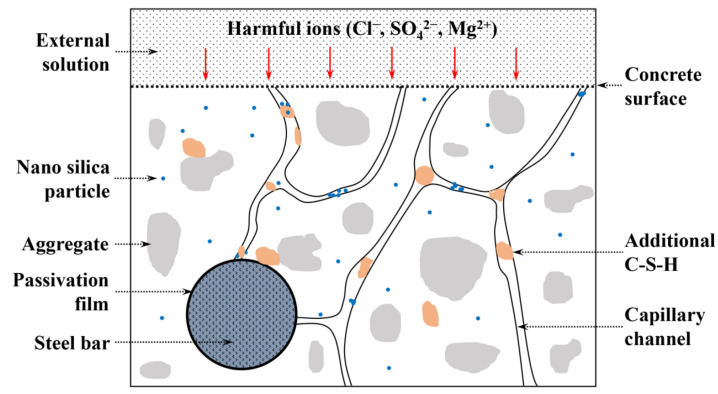
Schematic illustration of the enhancement mechanism of CNS.

The mechanism for refining the pore structure and improving the impermeability of concrete by incorporating CNS was interpreted as Figure 21. The harmful ions, such as chloride ion, penetrate into the matrix through the capillary channels in cement-based materials [43]. The passivation film would be oxidized gradually once chloride ion reaches the steel bar surface, which eventually leads to the corrosion and deterioration of the structure. The higher the chloride diffusion coefficient, the faster the corrosion rate of steel bar.

As in the analysis above, once in contact with water cement mineral ions were immediately dissolved and accompanied by the generation of various hydration products. When CNS was incorporated, the uniformly dispersed nano-silica particles could provide crystallization nucleation points for the hydration reaction, thus promoting the crystallization growth of the hydration products [30]. In addition, plenty of additional C-S-H gels were generated due to its high chemical reactivity, and these were responsible for the close interconnections between hydration products thus refining the pores and developing a discontinuous pore structure [44]. Meanwhile, large amounts of CH were consumed during this process and the size of the CH crystal was restricted. Moreover, owing to the micro-aggregate effect, nano-sized silica and C-S-H particles blocked the capillary channels in cement matrix. In this case, the pore size was refined and the inhomogeneity and complexity of the pore structure was enhanced. Consequently, a denser microstructure with more complex and discontinuous pore structure was obtained, and the external media (water and harmful ions) were more difficult to ingress the interior, thus decreasing the possibility of deterioration of hardened cement-based materials.

## 4. Conclusions

From the analysis above in this study, several conclusions can be obtained:

1. CNS promoted the early cement hydration process and consumed plenty of CH. The total exothermic heat of the sample with 3% CNS increased by 26.0% compared with the sample C0 at 72 h;
2. CNS improved the pore structure considerably: many of the micro pores converted into micro pores and the complexity of the spatial distribution of pores was increased as well. The porosity of N3 reduced from 0.1163 mL·g^−1^ to 0.1103 mL·g^−1^ compared to sample C0;
3. The incorporation of 3% CNS significantly decreased the Ca/Si ratio of ITZ, which reduced from 3.18 to 2.22 in comparison with sample C0. This made the ITZ more compact, helping to improve the strength and impermeability;
4. The impermeability of samples with CNS including water absorption, sorptivity coefficient, and chloride diffusion coefficient were significantly improved. Within a certain range (0–3%), the higher the CNS content, the better the improvement effect.

## Figures and Tables

**Table 1:** Chemical component of Portland cement.

Compound	Cement (wt.%)
SiO_2_	21.07
Al_2_O_3_	4.71
Fe_2_O_3_	3.21
SO_3_	2.10
CaO	63.06
MgO	1.78
Na_2_O	0.12
K_2_O	0.68
Loss on ignition (LOI)	2.42
Total	99.15

**Table 2:** Physical characteristics of CNS.

CNS	Solid Content (wt.%)	Average Particle Size (nm)	pH	Viscosity (Pa·s)	Density (g/cm^3^)
Indicator	40	14	10.3	4.7	1.21

**Table 3:** Mix proportion of mortar and paste.

Mixture	Cement (wt.%)	CNS (wt.%)	W/B	Sand (wt.%)
Mortars				
C0	100	0	0.5	300
N1	99	1	0.5	300
N2	98	2	0.5	300
N3	97	3	0.5	300
Pastes				
C0	100	0	0.5	
N1	99	1	0.5	
N2	98	2	0.5	
N3	97	3	0.5	

Note. All the CNS added was recorded as the solid content.

**Table 4:** Initial and secondary sorptivity coefficient of mortars.

Mix No.	CNS Dosage (%)	Initial Sorptivity Coefficient (mm/min^1/2^)	Secondary Sorptivity Coefficient (mm/min^1/2^)
C0	0	0.097	0.054
N1	1	0.067	0.042
N2	2	0.059	0.040
N3	3	0.053	0.033

**Table 5:** MIP results of 28 d cured samples C0 and N3.

Sample	Porosity (mL·g^−1^)	Pore Volume Distribution (mL·g^−1^)				
		6–10 nm	10–25 nm	25–50 nm	50–100 nm	>100 nm
C0	0.1163	0.0075	0.0262	0.0601	0.0133	0.0092
N3	0.1103	0.0154	0.0438	0.0342	0.0052	0.0117

**Table 6:** Quantitative analysis results of ^29^Si NMR spectra.

Sample	Relative Intensity of Q^n^/%			α_pc_/%	ACL
	Q^0^	Q^1^	Q^2^		
C0	29.54	60.8	9.66	70.45	2.32
N3	27.28	59.77	12.94	72.72	2.43

**Table 7:** Analysis of the element content of ITZ of different mortars.

	C0		N3	
	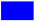	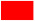	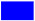	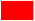
Area	Aggregate	Hydration Products	Aggregate	Hydration Products
Proportion (%)	27%	73%	19%	81%
Si (%)	40.67%	8.48%	43.15%	10.25%
Ca (%)	1.22%	26.95%	1.20%	22.78%
Ca/Si ratio of ITZ		3.18		2.22

## Data Availability

The data presented in this study are available on request from the corresponding authors.
